# A taxonomic study on the genus *Harpapion* Voss, 1966 from China (Coleoptera, Apionidae)

**DOI:** 10.3897/zookeys.358.6136

**Published:** 2013-12-04

**Authors:** Zhiliang Wang, Miguel A. Alonso-Zarazaga, Runzhi Zhang

**Affiliations:** 1 Key Laboratory of Zoological Systematics and Evolution, Institute of Zoology, Chinese Academy of Sciences, Beijing, People’s Republic of China; 2 Depto. de Biodiversidad y Biología Evolutiva, Museo Nacional de Ciencias Naturales (CSIC), José Gutiérrez Abascal, 2, E-28006Madrid, Spain

**Keywords:** Weevil, *Flavopodapion*, new species, new record, morphology, systematics, key

## Abstract

*Harpapion safranum* **sp. n.** and *Harpapion borisi* **sp. n.** are described and figured. *Harpapion vietnamense* (Korotyaev, 1987) is recorded as new for China. The genitalia andterminalia of *H. considerandum*, *H. coelebs* and*H. vietnamense* are redescribed and redrawn. The diagnostic characters of *Harpapion* are defined. A key to the known species of the genus *Harpapion* from China is provided. Affinities with the genus *Flavopodapion* Korotyaev, 1987 are discussed.

## Introduction

The apionid genus *Harpapion* was erected and initially placed as a subgenus of *Apiotherium* Beguin-Billecocq, 1905 by Voss (1966), and it was later treated as a genus of Apioninae *incertae sedis* by Alonso-Zarazaga and Lyal (1999). Recently, it was considered a member of the tribe Aspidapiini Alonso-Zarazaga, 1990 based on its elongate-triangular scutellum and the genitalia with apparently primitive traits (Alonso-Zarazaga 2011). Moreover, it was recognized as an Afrotropical and Arabian genus (Alonso-Zarazaga and Lyal 1999) until two species, *Harpapion coelebs* (Korotyaev, 1987) and *Harpapion vietnamense* (Korotyaev, 1985), respectively from China and Vietnam were identified as new records of *Harpapion* in Southeast Asia (Alonso-Zarazaga et al. 2011). Wanat (1990) redescribed and figured in detail the Eritrean species *Harpapion dongollanum* (Wagner, 1910), and then Alonso-Zarazaga et al. (2011) pointed out the diagnostic characters of *Harpapion* compared with *Pseudaspidapion* Wanat, 1990.

Recently, we obtained two more specimens of the monotypic species *Harpapion vietnamense*, as well as two series of specimens which are considered to be two new species of the genus *Harpapion* from South China. In describing the new species below, we have also documented and figured the genitalia of *Harpapion considerandum* (type species of *Harpapion*), *Harpapion coelebs* and*Harpapion vietnamense*.

## Materials and methods

Materials examined of the new species for this study are to be deposited in the Institute of Zoology, Chinese Academy of Sciences, Beijing (IZCAS) and the Biological Museum, Sun Yat-Sen University, Guangdong (BMSYU). Type and identified specimens were obtained from BMSYU, Natural History Museum (NHM), Zoological Institute (ZIN) on loan or belong to IZCAS.

Descriptions were made and photographs were taken with a Canon EOS 5D mounted on a Nikon SMZ1500. Extended focus images were generated with CombineZM and edited with Adobe Photoshop CS 5.0 when required. Microscopic slides were studied under a Leica DM 2500 microscope and photos were taken with a Nikon CoolPix P7100. Drawings were made from the original photographs by using the software Adobe Illustrator CS5.0, or directly by using a drawing tube attached to the microscope.

Scanning Electron Microscope (SEM) photos were taken with a FEI ESEM Quanta 450 device and the software xT microscope control. Specimens were cleaned by hair pencil and mounted on the mounting card directly.

Nomenclature of the rostral parts follows Alonso-Zarazaga (1989) and that of genitalia follows Alonso-Zarazaga (1990).

The dissecting method used follows Alonso-Zarazaga (1990). Abdomens were put into 10% NaOH for several hours until the inner tissues were digested, and the resultant structures were placed on a temporary microscope slide for examination.

After description, the genitalia and other parts of each specimen were placed in DMHF on an acetate card for long term conservation (Steedman 1958; Bameul 1990).

Labels are described as they are (in Chinese or Cyrillic), with pinyin romanization for Chinese and ISO 9:1995 for Russian, and translations in square brackets; labels are separated by semicolons and lines by slashes. Alternative modern pinyin romanizations are also given in some cases where labels are written using other romanization systems, like Wade-Giles.

## Taxonomic treatment

### *Harpapion* Voss, 1966

According to species examined, the diagnostic characters of *Harpapion* could be defined as follows: 1) scutellum triangular, distinctly elongate and basally projecting, apically raised (Figure 13); 2) the meso- and metatibial mucros are evidently elongate and bent at their apices (rather than short and straight in *Pseudaspidapion*) (Figures 14, 16); 3) the rostrum is clearly dilated at the antennal insertion and distinctly constricted apicad in dorsal view; 4) the setae fringing the front margin of pronotum are parallel to it; 5) the scales are broad, especially on the meso- and metarostrum, head and propleuron, etc. 6) the 1^st^ elytral stria at base reaches the middle level of the scutellum; 7) the pygidium is of the aspidapionine type, subsemicircular in dorsal view, with the apical flange strongly raised, the transverse sulcus deep but not cutting the sides; 8) the ninth sternite (spiculum gastrale) is Y-shaped, slender and subsymmetrical, not winged; 9) the apex of the penis is moderately to distinctly curved in lateral view, sometimes the pedon is recurved at apex; 10) the tegminal plate is articulated with the free ring, laterally developed, enveloping; the parameroid lobes present a usually well developed apical membranous area with a small apical notch, the basal sclerotized area has a medial sinuation in its front margin and bears 4-7 macrochaetae on each side; the fenestrae are present and variable; the prostegium is bidentate with two lateral projections (absent in *Harpapion coelebs*).

#### Harpapion considerandum

(Fåhraeus, 1871)

http://species-id.net/wiki/Harpapion_considerandum

Figures 1–7

##### Remarks.

*Pygidium* subsemicircular in dorsal view, 0.83× as long as wide, apical flange strongly raised, transverse sulcus deep (Figure 5–6).

**Figures 1–7.**
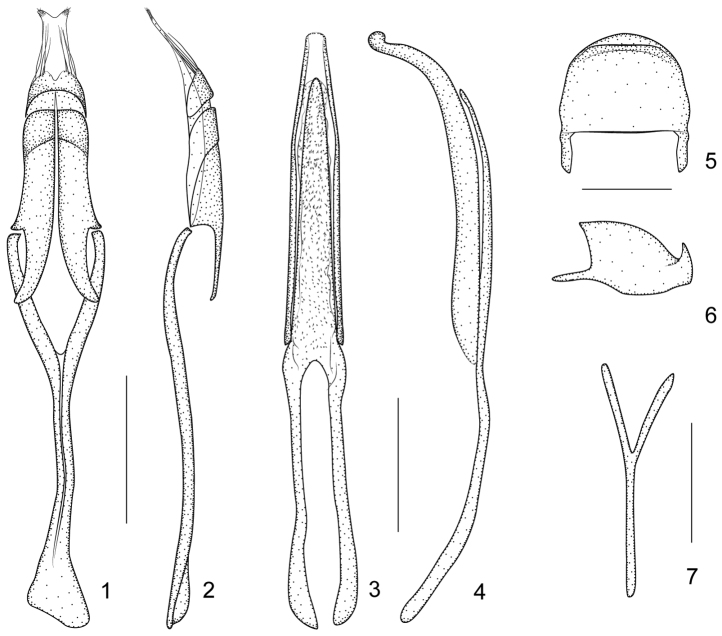
*Harpapion considerandum* Fåhraeus, 1871 **1** tegmen, dorsal view **2** tegmen, lateral view **3** penis, dorsal view **4** penis, lateral view **5** pygidium, dorsal view **6** pygidium, lateral view **7** ninth sternite (spiculum gastrale). Scales (mm): **1–2**: 0.2, **3–4**: 0.2, **5–6**: 0.1, **7**: 0.1.

##### Genitalia and terminalia.

Ninth sternite (spiculum gastrale) Y-shaped, slender and nearly symmetrical, not winged, manubrium about 1.21× as long as arms (Figure 7). Penis in dorsal view with pedon gradually and distinctly constricted apicad from apical 1/3, apex rounded, tectum apically constricted; temones moderately elongate, about 0.76× as long as pedon; in side view pedon extremely incurved at apical 1/4, nearly forming a 60° angle, with apical plate slightly recurved at extreme apex, without projections; endophallus with dense tiny spicules (Figures 3–4). Tegminal plate articulated with free ring, laterally developed, enveloping; parameroid lobes with apical membranous area long, bearing two tufts of visible microchaetae apically; basal sclerotized area elongate, front margin weakly sinuate medially, bearing 4 long macrochaetae dorsolaterally on each side; fenestrae large, not separated; linea arquata visible; prostegium bidentate, teeth elongate, curved; median unsclerotized strip elongate and nearly reaching the anterior margin of fenestrae. Manubrial apex asymmetrically broadened (Figures 1–2).

##### Materials.

1♂: (white, printed): Magila / E. Africa / A. V. Legros / 98-190; (white, printed and handwritten): Wagner det. ♂ / considerandum Fåhr.; 1♀: (white, handwritten): X. 1983 / in clove tree / CIEA15888; (white, handwritten): Tanzania / Zanjibar / Kitunda; (white, printed): pres by: Comm Inst Ent B. M. 1984-1. Specimens in the NHM.

##### Distribution.

*Harpapion considerandum* s. str. has been recorded from the following African countries: Guinea, Cameroon, Republic of the Congo, Democratic Republic of the Congo (ex Zaire), Chad, Ethiopia, Kenya, Tanzania, Uganda, Angola, Zimbabwe and South Africa (Eastern Cape, Western Cape and Natal) (Balfour-Browne 1944; Beguin-Billecocq 1910; Fåhraeus 1871; Hoffmann 1968a, 1968b; Hustache 1929, 1939; Marshall 1940; Voss 1959, 1966, 1974; Wagner 1911). Two subspecies, whose status is doubtful, have been recorded as follows: ssp. *circumscriptum* (Hartmann, 1897) from Senegal, Guinea, Mali, Nigeria and Tanzania (Hartmann 1897; Voss 1966); and ssp. *combustum* (Wagner, 1908) from Tanzania and Angola (Marshall 1953; Wagner 1908).

#### Harpapion coelebs

(Korotyaev, 1987)

http://species-id.net/wiki/Harpapion_coelebs

Figures 25–32

##### Remarks.

*Pygidium* subsemicircular in dorsal view, 0.83× as long as wide, apical flange strongly raised, transverse sulcus distinctly depressed and wide, disc with 2–3 rows of punctures between disc and sulcus, pubescent sparse and minute (Figures 29–30).

##### Genitalia and terminalia.

Eighth sternite transverse, with apical edge wide, slightly concave (Figure 31). Ninth sternite (spiculum gastrale) Y-shaped, very weak, almost symmetrical, not winged, manubrium about 1.83× as long as arms (Figure 32). Penis in dorsal view with sides almost parallel, apical plate ogival and distinctly constricted apicad in a short rectangular projection, with 2 subdentiform projections, tectum evidently constricted apicad, in side view, pedon depressed, moderately curved, apical plate slightly incurved; temones slim, about 0.43× as long as pedon; endophallus with markedly dense spicules (Figures 27–28). Tegminal plate articulated with free ring, moderately depressed, slightly enveloping laterally; parameroid lobes with apical membranous area long and tapering apicad, without microchaetae; basal sclerotized area short, front margin widely sinuate in middle, with 6 long macrochaetae and 8-10 sensilla on each side; fenestrae distinctly enlarged, transverse, narrowly separated; linea arquata visible; prostegium extremely concave without any projections; median unsclerotized strip elongate and surpassing fenestrae. Manubrial apex broken when dissected (the apex of manubrium should be tangled inside the thorax) (Figures 25–26).

##### Materials.

Paratype: 1♂: (white, printed): 云南景东董家坟 [Yúnnán JǐngDōng Dǒngjiāfén] / 1956.V.28/1250m / leg. 克雷让诺夫斯基 [Kryzhanovsky]; (white, printed): Юньнань, 10 км. N Цзин- [Yúnnán, 10 km north of Jǐng-] / дуна [-Dōng], 1250 м, 28. V. 1956 / Крыжановский [Kryzhanovsky].

##### Distribution.

Yunnan.

#### Harpapion vietnamense

(Korotyaev, 1987)

http://species-id.net/wiki/Harpapion_vietnamense

Figures 33–40

##### Remarks.

*Pygidium* subsemicircular in dorsal view, 0.89× as long as wide, apical flange strongly raised, transverse sulcus deep and narrow, disc nearly smooth, without visible punctures or pubescence (Figures 37–38).

##### Genitalia and terminalia.

Eighth sternite moderately elongate, apical edge constricted, relatively concave, basal edge with a medial tubercle, sides distinctly extended backwards (Figure 39). Ninth sternite (spiculum gastrale) Y-shaped, symmetrical, not winged, manubrium about 2.25× as long as arms (Figure 40). Penis in dorsal view with pedon sides almost parallel, apically elongate and distinctly constricted halfway, with 2 subdentiform projections, tectum evidently dilated apicad, in lateral view, pedon slightly depressed, apical plate distinctly elongate and gently curved ventrad, extreme apex recurved; temones about 0.74× as long as pedon; endophallus in anterior half with dense spicules and basal half with 2 sclerotized semicylindrical, hollow structures fused at base (Figures 35–36). Tegminal plate articulated with free ring, about 0.94× as long as manubrium, moderately depressed, slightly enveloping laterally; apical membranous area of parameroid lobes developed and extremely tapering apicad, with a minute apical notch, without distinctly visible microchaetae; basal sclerotized area slightly elongated, front margin widely and roundly sinuate, with 5–6 long macrochaetae and 8-10 sensilla on each side; fenestrae tranverse, not separated; linea arquata present; prostegium evidently bidentate, teeth elongate, almost straight, acute; median unsclerotized strip elongate and reaching the linea arquata. Manubrial apex distinctly broadened (Figures 33–34).

##### Materials.

Holotype. ♂ (white, handwritten): Вьетнам, пров. Хашонбинь [V’etnam, prov. Hašonbin’] [Vietnam, prov. Hà Sơn Bình] / 7 км ю.-в. Хоабиня, вторичный тропический лес и кустарник на склоне [7 km û.-v. Hoabinâ, vtoričnyj tropičeskij les i kustarnik na sklone] [7 km SE of Hòa Bình, tropical secondary forest and scrub on the hillside] / 17. X 1976 / leg. Л. Н. Медведев [L. N. Medvedev]; 1♂: 云南景洪勐海县纳板河 [Yúnnán Jǐnghóng Měnghǎixiàn Nàbǎnhé] / 保护区过门山（森林） [bǎohùqū Guòménshān (sēn lín [forest]) / 2009.V.06, 1114m; 22.24644°N, 100.60610°E, 飞阻 [Fēizŭ (flight intercept)] / 采集人：孟令曾 [Cǎijírén (leg.): Mèng Lìngzēng]; Guomenshan, VI/1D / 06.05.2009/ leg. L. Z. Meng; IOZ(E)1369311; 1♂: 云碧大开河 [Yún Bì Dàkāihé] / 1957.IV.23 / 朱增浩 [leg. Zhū Zēnghào]; IOZ(E)1639312.

##### Distribution.

Yunnan (new record for China), Vietnam.

#### Harpapion safranum

Wang & Alonso-Zarazaga sp. n.

http://zoobank.org/887BB3CE-2FF2-4337-A439-88763EA93AE1

http://species-id.net/wiki/Harpapion_safranum

Figures 13 , 14 , 41 –52

##### Description

(holotype). *Measurements* (in mm): Standard length: 1.84. Rostrum: length: 0.77, maximum width: 0.16. Pronotum: median length: 0.53, maximum width: 0.57. Elytra: median length: 1.33, maximum width: 0.92.

*Integument* (Figures 41–42) generally piceous, antennae, prorostrum and tarsi dark reddish brown, femora reddish brown, and tibiae pale reddish brown.

*Vestiture* composed of distinctly whitish, thick, lanceolate scales with acute to rounded apices, rarely truncate (some scales on hind margin of eyes elliptical to nearly rhombic) and grayish acute hairs on antennae, tibiae and tarsomeres. Pronotal vestiture centripetal, scales on apex parallel to margin, but on base perpendicular to margin, pronotal disc with scales distinctly longer and thicker than on legs, reaching base of anterior scales. Elytral scales in one row per interstriae, two irregular rows on disc, scales on striae 1/2-2/3 as long as scales on interstriae. One specialized seta on apical region of 9^th^ interstria.

*Rostrum* cylindrical and moderately robust, in dorsal view 8.25× as long as apical width, 1.45× as long as pronotum in midline, widest at mesorostrum, prorostrum constricted apicad, metarostrum with sides almost parallel, metarostrum with median dorsal area impunctate, dorsal submedial sulci and dorsal submedial keels weak, minutely punctate and pubescent, lateral area of metarostrum and prorostrum with weak oblong confluent punctures, weakly microreticulate, apical third of prorostrum almost impunctate, smooth and shining; in lateral view moderately curved, sides converging to apex, carinae and sulci weak, ventral medial keel fine and complete, ventral sublateral keels with dense line of scales under mesorostrum.

*Head* transverse, frons very weakly convex, as wide as metarostrum, constricted behind eyes, medial area impunctate and glabrous, lateral areas with irregular rows of punctures and scales, subocular keel not reaching middle of eyes, area between subocular keel flat, microreticulate and impunctate. Eyes round, moderately convex.

*Antennae* inserted at basal 0.23 of rostral length, scape 3.20× as long as wide, about as long as mesorostral width. Pedicel 2.00× as long as wide, as long as desmomeres 2+3, desmomeres 2–3 1.33× as long as wide, desmomeres 4–5 and 7 1.00× as long as wide, desmomere 6 0.75× as long as wide. Club oval, slightly flattened, 1.88× as long as wide, as long as last 5.5 desmomeres, sutures obsolete.

*Pronotum* campaniform, 0.93× as long as wide, apical constriction relatively strong, little wider at base than at middle, base 1.39× as wide as apex, bisinuate with rounded medial projection towards scutellum, basal flange developed. Prescutellar fovea shallow, very short, about as broad as diameter of one puncture, as long as 2–3 diameters, reaching 1/4 of pronotum. Discal punctures relatively deep, ca. 0.5–1× diameter apart, interspaces moderately convex, microreticulate.

*Scutellum* elongate, triangular, ca. 2.00× as long as wide, 2 basal tubercles fused at base in front view, apical tubercle rounded, slightly prominent and hardly visible in lateral view (Figure 13).

*Elytra* elongate, 1.45× as long as wide, 2.51× as long as pronotum, widest almost at middle, humeri moderately developed, striae deep, about as wide as interstriae, punctures elongate, apically connected 1+2+9, 3+4, 5+6, 7+8, interstriae evidently convex with small punctures, surface distinctly wrinkled, not microreticulate, shining.

*Ventral areas*. Mesocoxae separated by a distance of 0.17× own transverse diameter. Metasternum 0.88× as long as mesocoxae. Mesosternal apophysis more prominent than metasternal apophysis. Anterior metasternal rim present. Abdominal ventrites microreticulate, length ratio along midline: 32-16-4-6-17. Ventrites 1-4coarsely punctate, Ventrite 5 minutely punctate with median convexity. Suture I marked only on sides, erased in middle, distance from hind margin of metacoxae as long as ventrite 2. Ventrite 5semicircular, transverse, 0.41× as long as wide. Pygidium subsemicircular, 0.71× as long as wide, apical flange strongly raised with row of punctures and hairs; transverse sulcus deep; disc pubescent and punctured as that of ventrite 5 (Figures 49–50).

*Legs*. Profemora little larger than metafemora, slightly robust, 3.11× as long as wide, widest at middle, minutely punctate. Protibiae almost straight, 7.05× as long as wide (Figure 33). Protarsomere 1 1.93× as long as wide, protarsomere 2 1.25× as long as wide, protarsomere 3bilobed, 0.82× as long as wide, lobes narrow, onychium 2.75× as long as wide, projecting from lobes of tarsomere 3 for 0.55× its length. Meso- and metatibial mucrones distinctly elongate and bent at apices (Figure 14), mesotibial mucro ca. 0.50× as long as apical tibial width, metatibial mucro longer than mesotibial one, ca. 0.67× as long as apical tibial width. Tarsal claws with conspicuous obtuse basal teeth.

*Genitalia and terminalia*. Eighth sternite moderately elongate, constricted to narrow, truncate apical margin, basal margin with sides distinctly extended backward (Figure 51). Ninth sternite (spiculum gastrale) Y-shaped, not winged, manubrium ca. 3.00× as long as arms (Figure 52). Penis in dorsal view with pedon slightly widened at level of ostium, distinctly constricted apicad, apical plate ogival, apex with button-like prong, tectum with sides almost parallel, apically moderately constricted, in lateral view, pedon depressed, moderately curved, apical plate slightly incurved; temones about 0.50× as long as aedeagal tube; endophallus without obvious structures (Figures 47–48). Tegminal plate articulated with free ring, laterally enveloping,; apical membranous area of parameroid lobes undeveloped, only laterally visible, without microchaetae; basal sclerotized area extremely enlarged and extended apicad, triangular-shaped, with 5 short macrochaetae on each lateroapical edge, without sensilla; fenestrae short, transverse, narrowly separated; linea arquata present, very close to basal margin of fenestrae; prostegium bidentate, teeth acute, slightly curved; median unsclerotized strip elongate and surpassing fenestrae. Manubrial apex evidently and asymmetrically broadened (Figures 45–46).

*Variation*. Male paratypes. Measurements (mm, n=5): Standard length: 1.62–1.90. Rostrum: length: 0.61–0.75, maximum width: 0.14–0.16. Pronotum: median length: 0.47–0.57, maximum width: 0.51–0.60. Elytra: median length: 1.26–1.46, maximum width: 0.72–0.92. Female paratypes (Figures 43–44). Measurements (mm, n=2): Standard length: 1.801.86. Rostrum: length: 0.720.79, maximum width: 0.160.17. Pronotum: median length: 0.510.54, maximum width: 0.560.58. Elytra: median length: 1.441.42, maximum width: 0.900.91. Females differ from males by the rostrum entirely black without reddish apex, about 1.41–1.46× longer than pronotum, antennae inserted at basal 0.33 of rostrum, tibiae simple, unarmed. Otherwise practically as in male.

##### Type-locality.

China, Guangdong: Zhongshan City eighth district, 22°30'58.82"N, 113°23'36.81"E.

*Materials*. Holotype: 1♂: 广东中山八区 [Guǎngdōng Zhōngshān Bāqū] / 1957.VII.31; 中国科学院 [zhōngguókēxuéyuàn] / 山坡草地 [shānpōcǎodì] / 46; IOZ(E)1639313; Paratypes: 3♂: Kwangtung [Guǎngdōng] S. China / Loh Fau Shan, [Luófúshān], / Poh-lo [Bóluó] District / April 6–8, 1934 / K. C. Yeung; En-071380~En-071382; 1♂: Hong Kong: Un-long [Yuánláng], / New Territories / September 19. 1940 / J. Linsley Gressitt; En-071403; 1♂: Hainan I. South China / Ta-hian [probably Dàoxiǎng], Alt. 300 met. / N. side, 5- Finger Mts. [Wúzhĭshān] / VI-12-1935 / L. Gressitt / En-071423; 1♀: Kwangtung [Guǎngdōng], S. China / Ho-yün [Heyun] to Wui-lung [probably Wéilóng] / Ho-yuen [Héyuán] District / Apr. 7, 1940. J. L. / Gressitt & F. K. To; En-071409; 1♀: Kwangtung [Guǎngdōng], S. China. / Sin-fung [Xīnfēng] to Lung-kai [Longgai (probably Lóngjīe which can be found on the modern maps)]. / Sin-fung [Xīnfēng] & Lien-p’ing [Liánpíng] / Dist’s, Apr. 12, 1940 / L. Gressitt & F. E. To; En-071367.

##### Distribution.

Guangdong, Hong Kong, Hainan.

##### Type deposition.

Holotype will be deposited in IZCAS, while all paratypes in BMSYU.

##### Remarks.

*Harpapion safranum* sp. n., can be easily recognized from other species from China by its external characters (red colour of entire legs and antennae, etc.) However, it is extremely similar to *Harpapion considerandum* which can be distinguished from the former by genitalia which were described above and illustrated in Figures 1–7.

##### Etymology.

This species is named *safranum* after its testaceous legs. This is a Medieval Latin name of the plant now called *Crocus sativus* L. (saffron) which yields a yellowish-orange dye. It is considered a noun in apposition.

#### Harpapion borisi

Wang & Alonso-Zarazaga sp. n.

http://zoobank.org/03462889-EF2B-418A-BAE5-394758F1E2DB

http://species-id.net/wiki/Harpapion_borisi

Figures 53 –62

##### Description

(holotype). *Measurements* (in mm). Standard length: 1.22. Rostrum: length: 0.44, maximum width: 0.08. Pronotum: median length: 0.36, maximum width: 0.41. Elytra: length: 0.94, maximum width: 0.54.

*Integument* generally piceous, tibiae and tarsi relatively paler and antennae pale reddish brown (Figures 53–54).

*Vestiture* composed of whitish to semitransparent, partly thick, lanceolate scales with acute to rounded apices, and semi-transparent acute hairs on antennae, tibiae and tarsomeres. Head, meta- and mesorostrum bearing broader scales with rounded apices, prorostrum nearly glabrous. Pronotal vestiture centripetal, scales on apex parallel to margin, on base perpendicular to margin, pronotal sides with scales distinctly longer and thicker than those on disc and elytra, reaching base of anterior scales. Elytral scales in one regular row per interstria, scales on striae tiny, grayish to transparent. One specialized seta on apical region of 9^th^ interstria.

*Rostrum* cylindrical and moderately robust, in dorsal view 5.71× as long as apical width, 1.22× as long as pronotum in midline, widest at mesorostrum, prorostrum tapering apicad, tube-shaped, metarostrum slightly constricted at rostral base, metarostrum with no distinct sulci, two very shallow and parallel punctate dorsal submedial sulci expanded from mesorostral level to nearly middle of prorostrum, meso- and metarostrum surface microreticulate, matte, prorostrum smooth, shining, almost impunctate; in lateral view weakly curved, almost straight, sides converging to apex, each side with very thin low dorsal sublateral keel running from front margin of eye to upper margin of scrobe and beyond, limiting ventrally dorsal sublateral sulcus.

*Head* almost as long as wide, frons very weakly convex and slightly narrower than metarostrum, constricted behind eyes, medial area rough, wrinkled, subocular keels curving to meet medially, nearly reaching middle of eyes, area between subocular keel flat, microreticulate and impunctate. Eyes subcircular, distinctly convex.

*Antennae* inserted at basal 0.22 of rostral length, scape 2.5× as long as wide, about 0.71× as long as mesorostral width. Pedicel 1.75× as long as wide, nearly as long as desmomeres 2+3+4, desmomeres 2 2.0× as long as wide, desmomeres 3–7 ca. 1.00× as long as wide; club oval, slightly flat, 2.0× as long as wide, as long as the last 5.5 desmomeres, sutures obsolete.

*Pronotum* campaniform, 0.88× as long as wide, constriction relatively strong, at base nearly as wide as at middle, base 1.25× as wide as apex, bisinuate with rounded medial projection towards scutellum, basal flange developed. Prescutellar fovea shallow, puncture-like, elliptical, about 1.5× as broad as diameter of puncture, as long as 2 diameters. Discal punctures very shallow, weakly visible, ca. 0.5-1× diameter apart, interspaces moderately convex, microreticulate.

*Scutellum* large, elongate, triangular, ca. 2.5× as long as wide, with two separate basal tubercles, obtuse; apex constricted and slightly raised, moderately visible in lateral view.

*Elytra* elongate, 1.74× as long as wide, 2.61× as long as pronotum, widest almost at middle, humeri distinct, striae deep, about 0.50× as wide as interstriae, distinctly catenulate-punctate, punctures round to oblong, space between punctures about 2.0–3.0× as long as puncture length, apically connected 1+2+9, 3+4, 5+6, 7+8, interstriae evidently convex with wrinkled surface, not microreticulate, shining.

*Ventral areas*. Mesocoxae and metacoxae narrowly separated by distance of 0.21× and 0.30× of transverse diameter, respectively. Metaventrite 0.75× as long as mesocoxae. Mesoventral process slightly more prominent than metaventral process. Anterior metasternal rim almost absent. Abdominal ventrites microreticulate, with length ratios along midline: 22-14-4-5-13. Ventrites 1–2 coarsely punctate, 3–5 very sparsely and minutely punctate, ventrite 5 minutely punctate with median convexity. Suture I scarcely visible, distance from hind margin of metacoxae, as long as ventrite 2. Ventrite 5 subsemicircular, transverse, 0.39× as long as wide. Pygidium suboblong, 0.76× as long as wide, apical flange strongly raised with row of punctures and hairs; transverse sulcus distinctly depressed; disc pubescent and punctured as that of ventrite 5(Figures 59–60).

*Legs*. Profemora slightly larger than metafemora, slightly robust, 3.07× as long as wide, widest at middle, minutely punctate. Protibiae almost straight, 6.33× as long as wide. Protarsomere 1 2.4× as long as wide, protarsomere 2 0.86× as long as wide, protarsomere 3bilobed, 0.75× as long as wide, lobes narrow, onychium 2.0× as long as wide, projecting from lobes of tarsomere 3 for 0.50× its length. Meso- and metatibiae similarly mucronate, mucros incurved at apices. Tarsal claws with conspicuous, acute basal teeth.

*Genitalia and terminalia*. Eighth sternite short, apical edge relatively wide, distinctly concave (Figure 61). Ninth sternite (spiculum gastrale) Y-shaped, not winged, manubrium about as long as arms (Figure 62). Penis in dorsal view, with pedon sides almost parallel, apex distinctly constricted, apical plate subtriangular, with 2 small dentiform projections, tectum evenly constricted apicad; temones about 0.71× as long as aedeagal tube; in lateral view, pedon depressed, moderately curved, apical plate slightly incurved, dentiform projections visible; endophallus with dense minute spicules and basally with 2 elongate incurved structures (Figures 57–58). Tegminal plate articulated with free ring, slightly enveloping in lateral view; not notched apically, apical membranous area of parameroid lobes developed and tapering apicad, without microchaetae; basal sclerotized area short, apical margin deeply sinuate medially, with 5 long macrochaetae on each lateroapical edge, without sensilla; fenestrae short, transverse, narrowly separate; linea arquata present; prostegium bidentate, teeth short, narrow, acute; median unsclerotized strip absent. Manubrial apex moderately broadened near apex (Figures 55–56).

*Variation*. Male paratype. Measurements: Standard length: 1.20. Rostrum: length: 0.42, maximum width: 0.10. Pronotum: median length: 0.34, maximum width: 0.36. Elytra: median length: 0.96, maximum width: 0.50. Female unknown.

##### Type-locality.

China, Yunnan, Menghai, Nabanhe National Natural Reserve, Guomenshan, 22.24644°N, 100.60610°E.

##### Materials.

Holotype: 1♂: (white, printed): 云南景洪勐海县纳板河 [Yúnnán Jǐnghóng Měnghǎixiàn Nàbǎnhé] / 保护区过门山（森林） [bǎohùqū Guòménshān (sēn lín [forest])] / 2009.III.16 1114m; 22.24644°N, 100.60610°E飞阻 [Fēizŭ (flight intercept)] / 采集人：孟令曾 [Cǎijírén (leg.): Mèng Lìngzēng]; Guomenshan VI/1D / 16.03.2009 / leg. L. Z. Meng; IOZ(E)1639309; Paratype: 1♂, (white, printed): 云南景洪勐海县纳板河 [Yúnnán Jǐnghóng Měnghǎixiàn Nàbǎnhé] / 保护区过门山（森林） [bǎohùqū Guòménshān (sēn lín [forest])] / 2009.VI.26 1114m; 22.24644°N, 100.60610°E飞阻 [Fēizŭ (flight intercept)] / 采集人：孟令曾 [Cǎijírén (leg.): Mèng Lìngzēng]; Guomenshan VI/1D / 26.06.2009 / leg. L. Z. Meng; IOZ(E)1639310.

##### Distribution.

Yunnan.

##### Type deposition.

Both holotype and paratype will be deposited in IZCAS.

##### Remarks.

*Harpapion borisi* sp. n. can be distinguished from other congeners by the following traits: 1) body standard length 1.22–1.24 mm (the others more than 1.5 mm); 2) elytral scales with similar size, in one regular row per interstria; 3) rostral pubescence not surpassing middle of prorostrum, nearly entire prorostrum glabrous; 4) prostegial teeth short and narrow; 5) tegminal median unsclerotized strip absent; 6) spiculum gastrale with manubrium about as long as arms.

##### Etymology.

This species is named after the Russian curculionidologist Boris A. Korotyaev, who has much improved the taxonomy of Apionidae from South China and helped us in many ways.

### Key to species of *Harpapion* from China (based on male characters)

**Table:** 

1	All legs and antennae reddish brown; apical plate of pedon without dorsal dentiform projections; fenestrae narrowly separated, transverse, rectangular; parameroid lobes with basal sclerotized area extremely enlarged and extended medially, triangular, apical membranous area reduced to lateral strip; median unsclerotized strip elongate and surpassing fenestrae	*Harpapion safranum* sp. n.
–	All legs black; apical plate of pedon with 2 dorsal dentiform projections; fenestrae separated or confused, differently shaped; parameroid lobes with basal sclerotized area short and medially sinuate, apical membranous area developed; median unsclerotized strip just or not reaching linea arquata	2
2	Body larger, standard length more than 2 mm; scales of elytral striae short, tips of anterior scales not or hardly reaching the base of posterior ones; antennal scape at least 3.5× as long as wide; tegmen with prostegium reduced and deeply concave, untoothed	*Harpapion coelebs*
–	Body smaller, standard length less than 2 mm; scales of elytral striae elongate, tips of anterior scales surpassing base of posterior ones; antennal scape at most 3.1× as long as wide; tegmen with prostegium developed and bidentate	3
3	Standard length 1.78–1.90 mm; rostral pubescence surpassing middle of prorostrum, only apex glabrous; elytral scales around scutellum distinctly whiter and thicker compared with semitransparent and thin scales on elytral disc; fenestrae fused; prostegium with very long teeth	*Harpapion vietnamense*
–	Standard length 1.22–1.24 mm; rostral pubescence not surpassing middle of prorostrum, nearly entire prorostrum glabrous; almost all elytral scales with same size and coloration; fenestrae separate; prostegium with short narrow teeth	*Harpapion borisi* sp. n.

## Discussion

Some previously considered generic characters of the tegmen like parameroid lobes, fenestrae and prostegium (Alonso-Zarazaga 1983, 1990) show a great amount of variation among the above species. The basal sclerotized area (the dorsal layer of the tegmen (Wanat 2001)) of parameroid lobes of the type species *Harpapion considerandum* is short and medially sinuate, and the apical membranous area (the ventral layer of the tegmen (Wanat 2001)) is well developed and prominent. These characters are also present in *Harpapion vietnamense*, *Harpapion coelebs* and *Harpapion borisi* but not in *Harpapion safranum* which externally resembles *Harpapion considerandum* very much. Also the fenestrae of *Harpapion considerandum* join medially, as in *Harpapion vietnamense*, but they are separated in the other species as well as in *Harpapion dongollanum*. Particularly, the prostegium of *Harpapion coelebs* is extremely retracted and lacks the lateral teeth, which is unique hitherto in this genus. After all, significant similarities of external characters as well as most internal characters let us conclude that they should remain for the time being in *Harpapion*, and the inconsistency mentioned above has to be considered as a specific divergence not having generic value.

**Figures 8–11.**
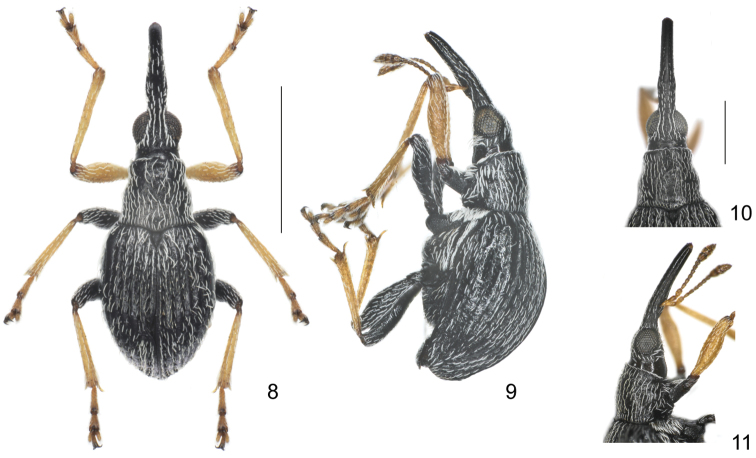
*Flavopodapion gilvipes* (Gemminger, 1871) **8** male, dorsal view **9** male, lateral view **10** female, head and rostrum, dorsal view **11** female, head and rostrum, lateral view. Scales (mm): **8–9**: 1.0, **10–11**: 0.5.

**Figures 12–16.**
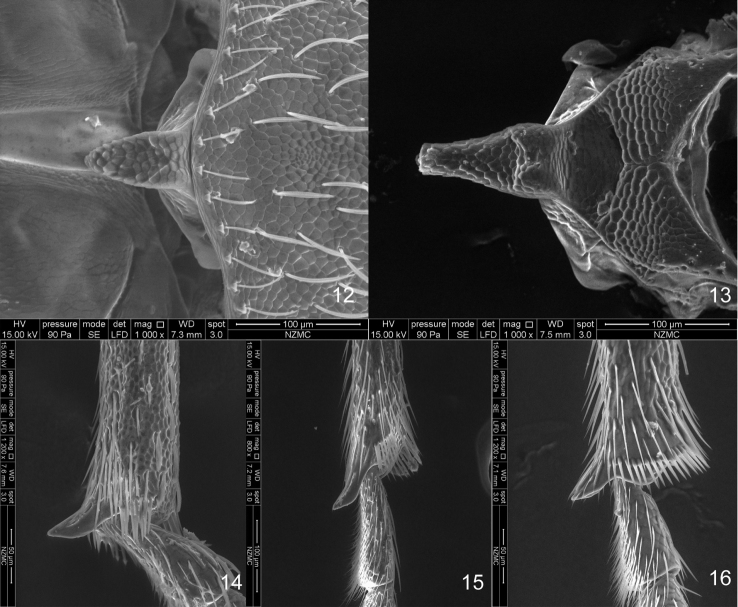
SEM photos **12–13** scutellum **12** *Flavopodapion gilvipes* (Gemminger, 1871) **13** *Harpapion safranum* sp. n. **14–16** metatibial mucro **14** *Harpapion safranum* sp. n. **15** *Flavopodapion gilvipes* (Gemminger, 1871) **16** *Pseudaspidapion botanicum* Alonso-Zarazaga & Wang, 2011.

**Figures 17–24.**
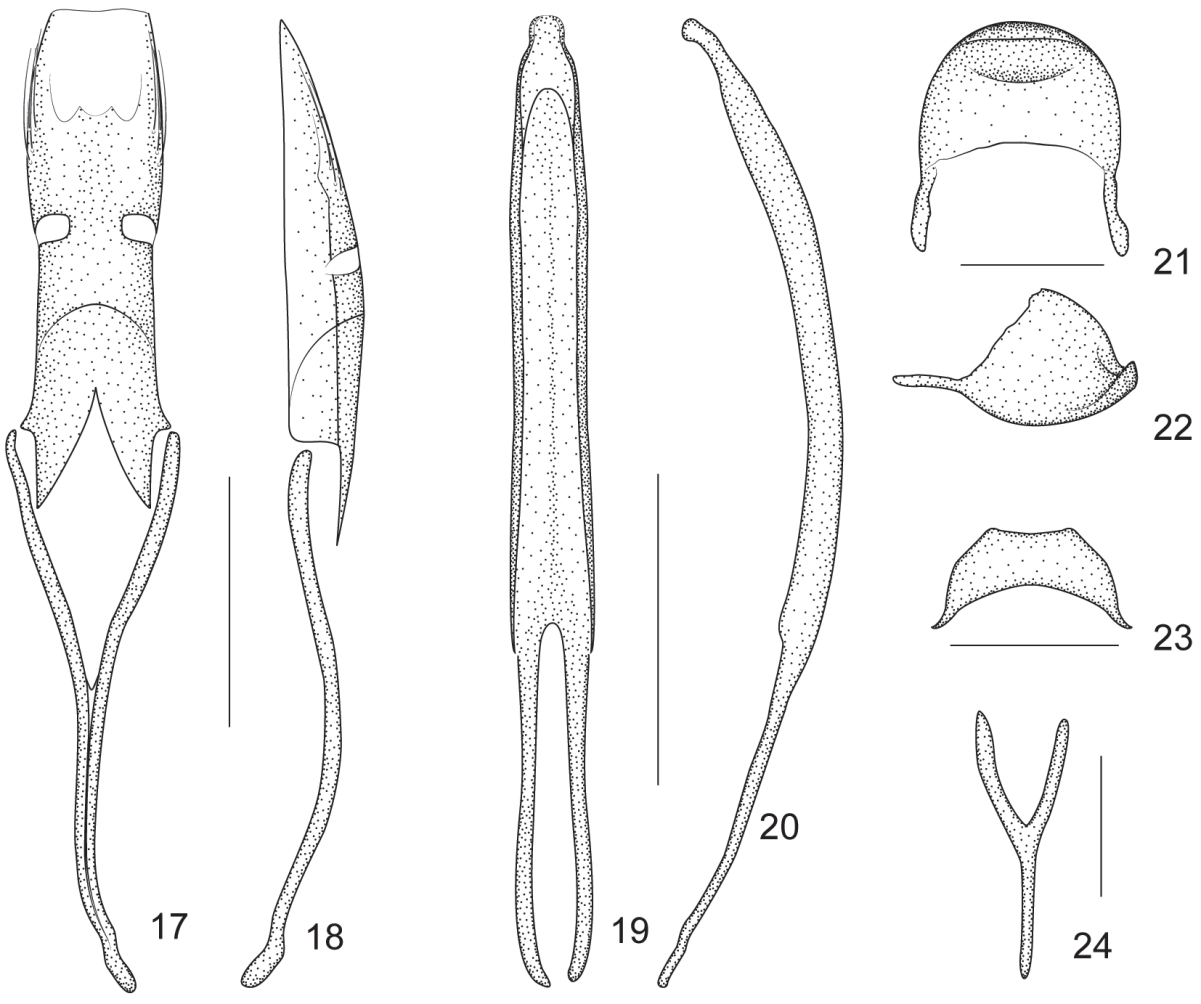
*Flavopodapion gilvipes* (Gemminger, 1871) **17** tegmen, dorsal view **18** tegmen, lateral view **19** penis, dorsal view **20** penis, lateral view **21** pygidium, dorsal view **22** pygidium, lateral view **23** eighth sternite, dorsal view **24** ninth sternite (spiculum gastrale). Scales (mm): **17–18**: 0.2, **19–20**: 0.2, **21–22**: 0.1, **23**: 0.1, **24**: 0.1.

Additionally, a close genus, *Flavopodapion* Korotyaev, 1987 (type species*Flavopodapion gilvipes* (Gemminger, 1871)) (Alonso-Zarazaga et al. 2011) resembles *Harpapion* very much. On one hand, its scutellum seems normally triangular, but two slightly raised basal projections can be found under SEM (Figure 12). On the other hand, its meso- and metatibial mucros show the peculiar bending present in the species of *Harpapion* (Figure 15). Other characters such as the rostrum clearly dilated at antennal insertion, the scale arrangement on the anterior and posterior margins of the pronotum and the apical flange of the pygidium strongly raised, etc. (Figures 8–11) coincide with those of *Harpapion*. However, some characters underline the differences between both genera, namely, in *Flavopodapion*, the relatively slender body, the triangular scutellum not constricted in the apical half and lacking a raised apex and clear basal projections. Moreover, the genitalia and terminalia show more significant differences: the ninth sternite (spiculum gastrale) with the manubrium distinctly shorter than the arms (Figure 24), the parameroid lobes with the basal sclerotized area evidently elongate, laterally extending apicad and leaving a membranous asetose area extending between the lobes and not tapering apicad, so that the apex in a general outline seems truncate, not pointed, the widely separate, reduced, lateral fenestrae, almost round in dorsal view, and the obsolete median unsclerotized strip (Figures 17–18). However, not too many related species are known in detail to allow drawing definite conclusions on the systematics of this particularly complex group of Apionidae.

**Figures 25–32.**
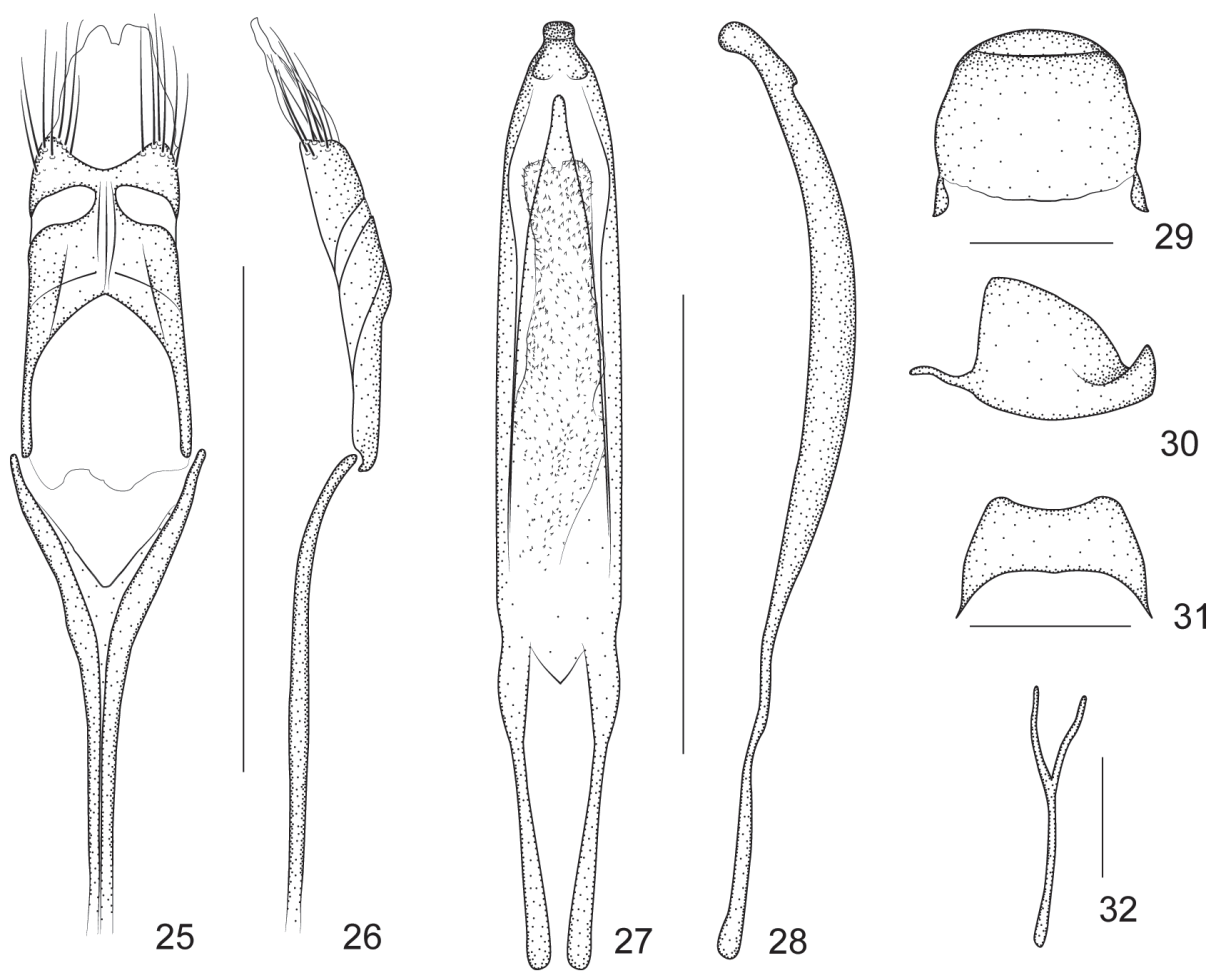
*Harpapion coelebs* (Korotyaev, 1987) **25** tegmen, dorsal view **26** tegmen, lateral view **27** penis, dorsal view **28** penis, lateral view **29** pygidium, dorsal view **30** pygidium, lateral view **31** eighth sternite, dorsal view **32** ninth sternite (spiculum gastrale). Scales (mm): **25–26**: 0.5, **27–28**: 0.5, **29–30**: 0.2, **31**: 0.2, **32**: 0.1.

**Figures 33–40.**
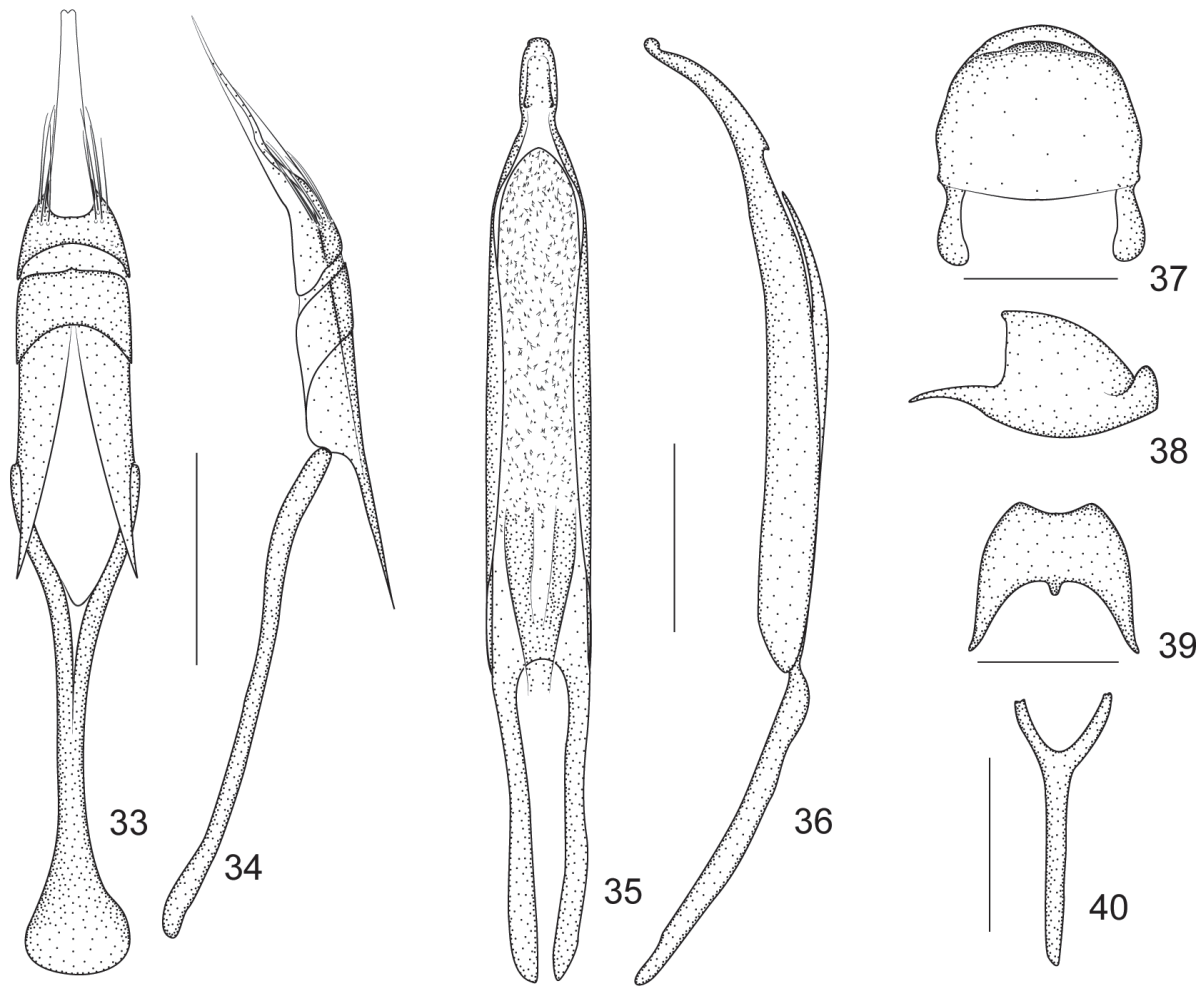
*Harpapion vietnamense* (Korotyaev, 1987) **33** tegmen, dorsal view **34** tegmen, lateral view **35** penis, dorsal view **36** penis, lateral view **37** pygidium, dorsal view **38** pygidium, lateral view **39** eighth sternite, dorsal view **40** ninth sternite (spiculum gastrale). Scales (mm): **33–34**: 0.2, **35–36**: 0.2, **37–38**: 0.2, **39**: 0.2, **40**: 0.2.

**Figures 41–44.**
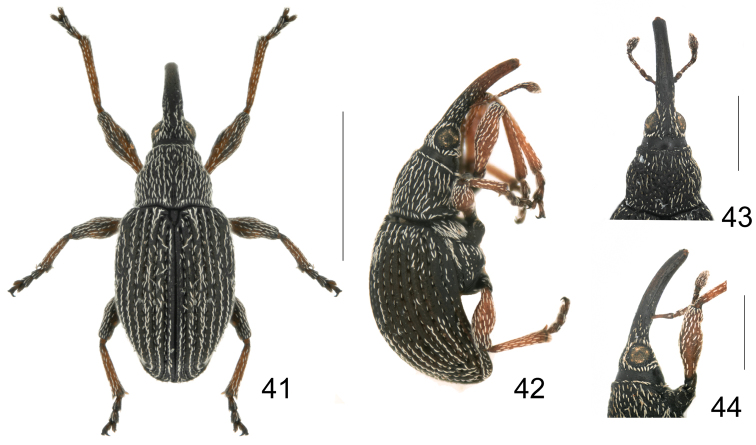
*Harpapion safranum* sp. n. **41** male, dorsal view **42** male, lateral view **43** female, head and rostrum, dorsal view **44** female, head and rostrum, lateral view. Scales (mm): **41–42**: 1.0, **43–44**: 0.5.

**Figures 45–52.**
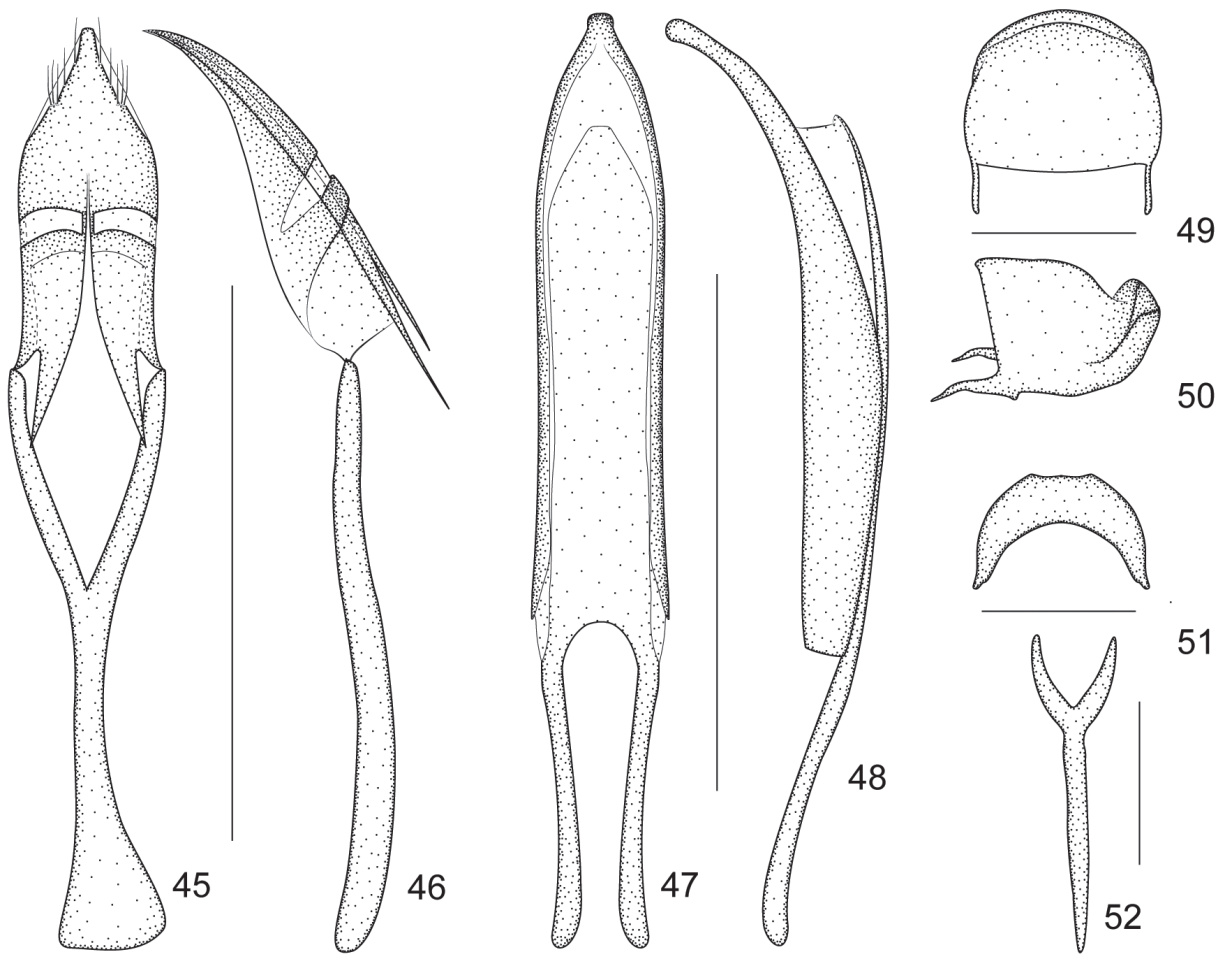
*Harpapion safranum* sp. n. **45** tegmen, dorsal view **46** tegmen, lateral view **47** penis, dorsal view **48** penis, lateral view **49** pygidium, dorsal view **50** pygidium, lateral view **51** eighth sternite, dorsal view **52** ninth sternite (spiculum gastrale). Scales (mm): **45–46**: 0.5, **47–48**: 0.5, **49–50**: 0.2, **51**: 0.2, **52**: 0.2.

**Figures 53–54.**
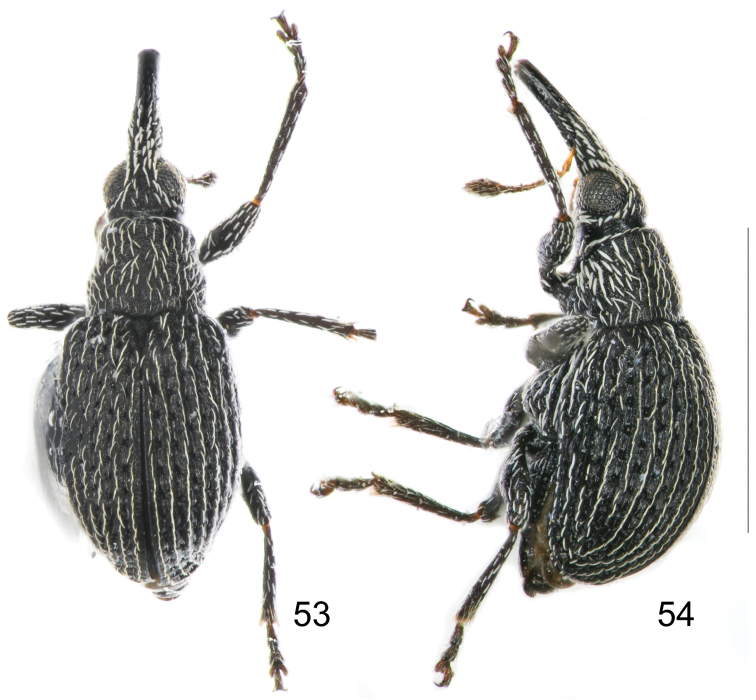
*Harpapion borisi* sp. n. **53** male, dorsal view **54** male, lateral view. Scales (mm): **53–54**: 1.0.

**Figures 55–62.**
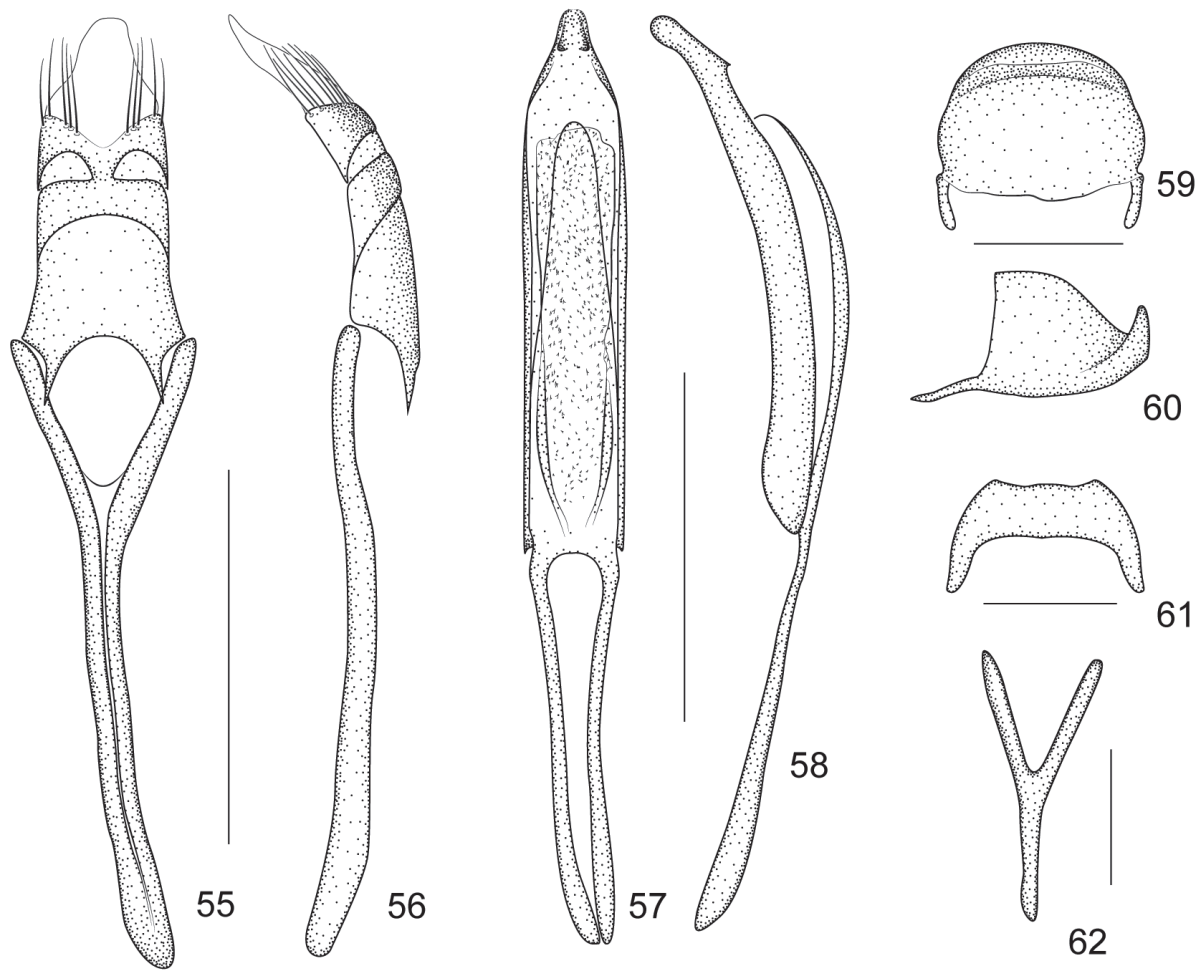
*Harpapion borisi* sp. n. **55** tegmen, dorsal view **56** tegmen, lateral view **57** penis, dorsal view **58** penis, lateral view **59** pygidium, dorsal view **60** pygidium, lateral view **61** eighth sternite, dorsal view **62** ninth sternite (spiculum gastrale). Scales (mm): **55–56**: 0.2, **57–58**: 0.2, **59–60**: 0.1, **61**: 0.1, **62**: 0.05.

## Supplementary Material

XML Treatment for Harpapion considerandum

XML Treatment for Harpapion coelebs

XML Treatment for Harpapion vietnamense

XML Treatment for Harpapion safranum

XML Treatment for Harpapion borisi
